# Bioluminescent RAPPID Sensors for the Single-Step Detection of Soluble Axl and Multiplex Analysis of Cell Surface Cancer Biomarkers

**DOI:** 10.1021/acs.analchem.2c00297

**Published:** 2022-04-19

**Authors:** Eva A. van Aalen, Simone F. A. Wouters, Dennis Verzijl, Maarten Merkx

**Affiliations:** † Laboratory of Chemical Biology, Department of Biomedical Engineering, Eindhoven University of Technology, P.O Box 513, 5600 MB Eindhoven, The Netherlands; ‡ Institute for Complex Molecular Systems, Eindhoven University of Technology, P.O Box 513, 5600 MB Eindhoven, The Netherlands; § Genmab, 3584 CT Utrecht, The Netherlands

## Abstract

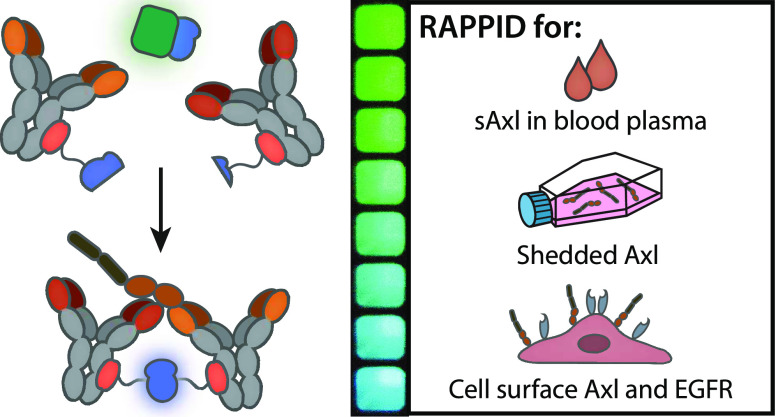

Early diagnosis of cancer is essential for the efficacy of treatment. Our group recently developed RAPPID, a bioluminescent immunoassay platform capable of measuring a wide panel of biomarkers directly in solution. Here, we developed and systematically screened different RAPPID sensors for sensitive detection of the soluble fraction of Axl (sAxl), a cell surface receptor that is overexpressed in several types of cancer. The best-performing RAPPID sensor, with a limit of detection of 8 pM and a **>**9-fold maximal change in emission ratio, was applied to measure Axl in three different contexts: clinically relevant sAxl levels (∼0.5 and ∼1 nM) in diluted blood plasma, proteolytically cleaved Axl in the cell culture medium of A431 and HeLa cancer cells, and Axl on the membrane of A431 cells. We further extended the sensor toolbox by developing dual-color RAPPID for simultaneous detection of Axl and EGFR on A431 and HeLa cells, as well as an AND-gate RAPPID that measures the concurrent presence of these two cell surface receptors on the same cell. These new RAPPID sensors provide attractive alternatives for more laborious protein detection and quantification methods such as FACS and immunostainings, due to their simple practical implantation and low intrinsic background signal.

## Introduction

Biomarker-specific point-of-care (POC) tests that enable noninvasive diagnostic testing and screening outside the hospital and traditional laboratories represent a promising approach for the diagnosis of early-stage cancer.^1,2^ Hepatocellular carcinoma (HCC) is the most common liver malignancy and early detection and prognosis increase therapy effectiveness.^3−5^ Therapeutic curative approaches, like surgery and chemotherapy, are typically only effective for early-stage HCC and limited for later stage of the disease.^6^ At present, imaging techniques such as transabdominal ultrasonography (US) are the most commonly used screening methods for high-risk patients.^7−9^ US is cost-effective but suboptimal for the detection of early-stage HCC, due to a moderate sensitivity of around 60%.^10^ The serum biomarker α-fetoprotein (AFP) is also used to detect early-stage HCC,^11,12^ but its low sensitivity (41–65%) makes it ill-suited for a POC diagnostic setting.^13^ Therefore, novel noninvasive serological biomarkers would greatly improve the early detection and prognosis of HCC and might enable the development of POC tests.

**Figure 1:**
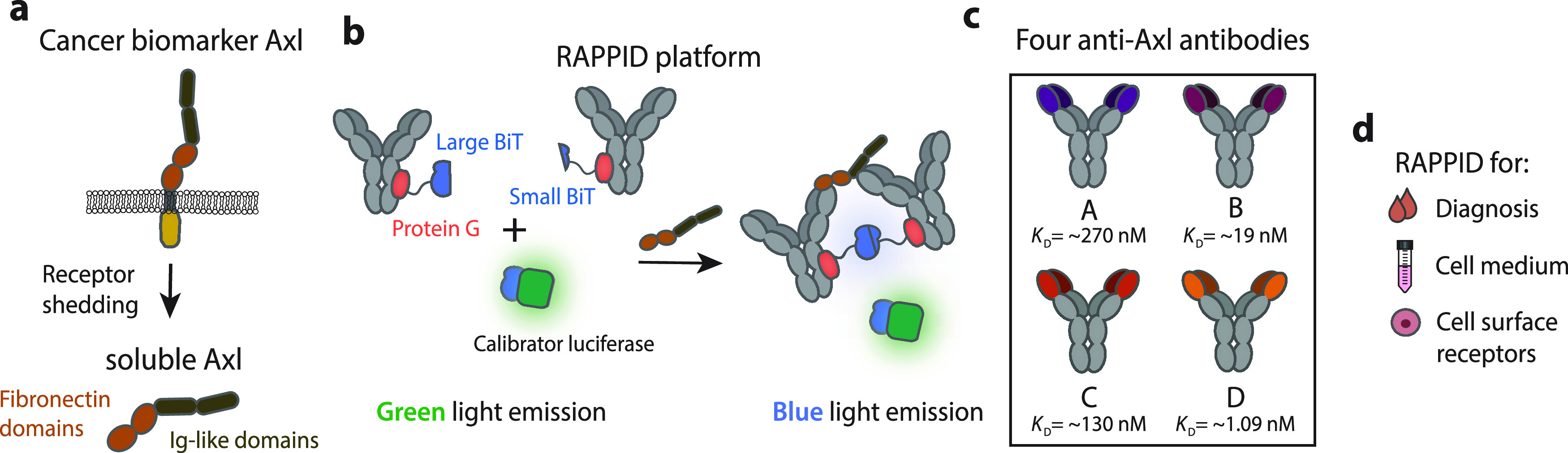
Development of RAPPID assays for detection of soluble Axl (sAxl). (a) Axl is overexpressed on the cellular membrane of various types of cancers. Shedding of Axl results in the release of the soluble extracellular fraction of Axl, which is subsequently found in blood plasma and can serve as a biomarker for the early diagnosis of hepatocellular carcinoma (HCC). (b) Schematic overview of the RAPPID assay. Anti-Axl antibodies are conjugated to either large BiT (LB) or small BiT (SB), the split variant of the NanoLuc luciferase (NLuc). Analyte binding results in the complementation of split NLuc, increasing the emission of blue light. The green light-emitting calibrator luciferase is used to make the RAPPID assay ratiometric, enabling accurate quantification of Axl directly in solution. (c) Four anti-Axl antibodies, with different affinities and epitopes, were used to develop six Axl-RAPPID variants. (d) The Axl-RAPPID assay is applied for diagnostic purposes, measurements in cell culture medium and for the detection of cell surface receptors.

Recent studies have shown that Axl is an accurate biomarker for early HCC and outperforms AFP.^14−16^ Aberrant expression of Axl, a member of the TAM (Tyro3, Axl, Mer) receptor family of the receptor tyrosine kinases (RTKs), is associated with various cancers, including renal cell carcinoma,^17^ non-small-cell lung cancer,^18−20^ breast cancer,^21^ melanoma,^22^ and HCC.^23^ The Axl receptor consists of an extracellular portion, with two fibronectin type III-like (FNIII-like) domains and two immunoglobulin-like (Ig-like) repeats, and an intracellular element with a tyrosine kinase domain.^24^ The activation and dimerization of Axl occurs via extracellular binding to its ligand growth arrest-specific gene 6 (Gas6) or via auto-activation as a result of Axl overexpression.^23,25^ Subsequent autophosphorylation and transphosphorylation of the intracellular domain of Axl induces downstream activation of pathways that promote cancer cell proliferation, invasion, migration, and survival.^23^ Furthermore, the receptor can be proteolytically cleaved or shedded, releasing an ∼80 to 85 kDa extracellular domain, known as soluble Axl (sAxl), which can be measured in blood plasma (Figure 1a).^26^ However, a challenge of using sAxl as a biomarker is the relatively small difference between serum sAxl concentrations in healthy individuals (40 ng/mL or ∼0.5 nM) and sAxl levels associated with early HCC (80 ng/mL or ∼1 nM) or late HCC (114.5 ng/mL or ∼1.43 nM).^14^ Currently, sAxl is often measured with ELISA,^14,27,28^ which requires multiple washing and incubation steps and is hence time-consuming, unsuitable for measurements directly in solution, and challenging to translate to POC applications. Current POC immunoassay formats such as lateral flow immunoassays (LFIA) do not allow accurate determination of biomarker concentration and can therefore not distinguish between the relative small differences in physiological and pathophysiological sAxl concentrations. A single-step detection method for sAxl that can be applied directly in blood plasma shows potential as a diagnostic tool for the early detection of HCC.

Bioluminescent-based homogeneous sensors that display a change in color upon analyte binding show great promise for measurements in complex media such as blood plasma, as minimal sample pretreatment is required.^29^ Unlike fluorescence-based methods, bioluminescent sensors do not need external excitation, thus eliminating issues associated with autofluorescence or light scattering.^29,30^ Recently, we established RAPPID (Ratiometric Plug-and-Play ImmunoDiagnostics), a mix-and-measure immunoassay platform based on the reconstitution of antibody-conjugated split NanoLuc luciferases.^31,32^ The platform is highly modular, as it entails monoclonal antibodies and photoconjugation through a protein G adaptor (Gx, Figure 1b).^33^ The straightforward development of a RAPPID assay enables the easy exchange of antibodies and hence screening for the best antibody pair and optimal sensor. Furthermore, the RAPPID platform has a high intrinsic maximal change in emission ratio and a robust ratiometric light output, enabled by the introduction of a green-emitting calibrator luciferase, facilitating the accurate detection of biomarkers in the picomolar to nanomolar range.^32^ The ratiometric nature of the RAPPID assay should make it an attractive diagnostic tool to detect challenging biomarkers, such as sAxl, with a small difference in concentrations associated with healthy and diseased individuals.

Here, four different human monoclonal antibodies, targeting either the FNIII-like or Ig-like domain of Axl, were utilized to develop six different RAPPID sensors and were systematically screened to obtain an optimal assay for sAxl detection (Figure 1b,c). This optimized assay was subsequently used to accurately quantify physiological and pathophysiological sAxl concentrations in blood plasma. We also further extended the RAPPID assay platform to enable both the direct detection of cell surface-bound Axl and the simultaneous single-step detection of multiple cancer-related cell surface receptors (Figure 1d).

## Experimental Section

### Cloning

The pET28a(+) vectors with DNA encoding for Gx-SB, Gx-LB, and calibrator luciferase (mNG-NL)^34,35^ were ordered from Genscript as described in ref (32). A pETa(+) expression plasmid for Gx-mNeonGreen-SB (Gx-mNG-SB) was developed by cloning DNA encoding for mNG into the Gx-SB vector by overhang extension PCR. The cloning results were confirmed by Sanger sequencing (BaseClear). DNA and amino acid sequences of Gx-SB and Gx-LB are listed in ref (32). DNA and amino acid sequence for Gx-mNG-SB can be found in Figure S1.

### Protein Expression

Gx-SB, Gx-LB, and Gx-mNG-SB were expressed as described before.^32^ The pEVOL-*p*BpF vector for the incorporation of the unnatural amino acid *p*-benzoylphenylalanine (*p*BpA, Bachem, 104504-45-2) was a gift from Peter Schultz (Addgene plasmid # 31190).^36^ All fusion proteins were purified using Ni^2+^ affinity chromatography followed by Strep-Tactin purification (IBA Lifesciences). The purity of Gx-SB, Gx-LB, Gx-mNG-SB, and calibrator luciferase was determined using SDS-PAGE analysis (Figure S2 and ref (32)). The human anti-Axl antibodies A, C, and D were generated as described in ref (22). Axl-B is an in-house produced prior-art antibody and is an IgG1 variant of the U3-11B7 antibody against Axl (U3 Pharma, WO 2009062690) with rat VH and human HC. All proteins were stored at −80 °C until use.

### Photoconjugation

Gx-SB and Gx-LB were photoconjugated to anti-Axl antibodies (A, B, C, and D), Gx-LB was coupled to cetuximab (obtained via the Catherina hospital pharmacy in Eindhoven) and Gx-mNG-SB was conjugated to cetuximab and anti-Axl C. Photoconjugation was performed for 90 minutes under a Promed UVL-30 UV light source (4 × 9 W) in PCR tubes in PBS (pH 7.4) on ice. The extent of photoconjugation was verified using a nonreducing SDS-PAGE analysis. An extensive photoconjugation protocol can be found in ref (32). Antibody conjugates were stored at 4 °C until use.

### Luminescent RAPPID Assays

RAPPID assays were done in PBS (pH 7.4, 0.1% (w/v) BSA) or diluted human blood plasma (ACD, DivBioScience) in nontreated white Thermo Scientific 384-well plates (Cat. no 262360) in a volume of 20 μL. An assay mix with 1–20 pM calibrator luciferase, 1 nM antibody-SB, and 1 nM antibody-LB was added to the buffer or diluted plasma, followed by an incubation step of 1 h at room temperature. After the addition of 1500-fold diluted (measurements in buffer) or 400-fold diluted (blood plasma measurements) NLuc substrate (Promega, N1110), the luminescent spectra were recorded between 398 and 653 nm on a Tecan Spark 10 M plate reader (bandwidth 25 nm; 22 °C). The blue-to-green ratios were calculated by dividing the blue light emission at 458 by the green light emission at 518 nm. The LOD was calculated using eq 1, in which SD is the standard error of the *y*-intercept, by linear regression of the blue-to-green ratios related to a selection of low sAxl concentrations.
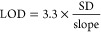
1

### RAPPID Assay in Cell Medium

HeLa cells and HEK293 cells were grown in Dulbecco’s modified Eagle’s medium (DMEM, from Gibco) and A431 cells were grown in RPMI medium (Gibco). All cells were cultured in Falcon corning T75 culture flasks (REF 353136). Both RPMI and DMEM were supplemented with 4.5 g/L d-glucose, 0.58 g/L l-glutamine, 10% fetal bovine serum (FBS), 100 U/mL penicillin, and 100 μg/mL streptomycin (all from Life Technologies), and grown at 37 °C with 5% CO_2_. Both DMEM and RPMI medium contained phenol red. The cells were cultured for three days until a confluency of 80% was reached. Subsequently, the medium (12 mL) was removed from the cells, centrifuged at 10,000*g* to remove residual cells, and diluted five-fold with PBS (pH 7.4, 0.1% (w/v) BSA). Next, the sensor mix (0.6 nM anti-Axl-C antibody-SB, 0.6 nM anti-Axl-D antibody-LB, and 2 pM calibrator luciferase) was added to the medium in a white Thermo Scientific 96-well plate (Cat. no 236108) to create a final culture medium concentration of 10%, in a total volume of 100 μL. After 1 h incubation, 1500× diluted NLuc substrate was added to the samples and bioluminescence was measured using a Tecan Spark 10 M plate reader.

### Cellular Assays

HeLa, HEK293, and A431 cells were cultured as described above. The cells were released from the culture flask using trypsin (Thermo Fisher Scientific) and counted with a digital cell counter (CytoSMART Cell Counter, version 3). Subsequently, the cells were washed with PBS (pH 7.4) and diluted to 5 million cells/mL in PBS (pH 7.4, 0.1% (w/v) BSA). An increasing number of cells was added to the sensor mixture (0.6 or 0.25 nM of antibody-sensor conjugate and 2 or 5 pM calibrator luciferase) in a 96-well plate to make a final volume of 100 μL. After the addition of 1500-fold diluted NLuc substrate, bioluminescence was monitored on a Tecan Spark 10 M plate reader with an integration time of 200 ms.

## Results and Discussion

### Development of sAxl-RAPPID Assays

The performance of the RAPPID platform is dependent on the antibodies used, as their affinities and the specific sandwich complex architecture determine the limit of detection (LOD), the detectable analyte concentration range, and the maximal change in emission ratio. Therefore, the antibody selection procedure is an important aspect of designing a RAPPID sensor. This is particularly critical when the physiologically relevant concentrations are in the picomolar to low nanomolar range and hence dilution of the sample, to allow tunable measurements of the analyte across the desired concentration range, is not obvious. Hence, to allow accurate distinction between healthy sAxl levels and concentrations associated with early HCC, we explored four previously developed human anti-Axl IgG1 antibodies (A, B, C, and D) with different antigen-binding properties.^22^ Mapping of the binding sites of these four antibodies revealed that antibodies A and B bind to the Ig2 domain of Axl and antibodies C and D to the FN1 and FN2 domain of the FNIII-like repeat, respectively (Table 1). Furthermore, the two Ig-like binders have overlapping binding sites on Axl, probably prohibiting the use of these two antibodies in one RAPPID assay. The affinities of the anti-Axl antibodies were determined using surface plasmon resonance (SPR), yielding dissociation constants (*K*_D_) in the low-high nanomolar range, ∼1.09, ∼19, ∼130, and ∼270 nM for anti-Axl D, B, C, and A, respectively (Figure S3 and Table 1).

**Table 1:** Overview of the Different sAxl-RAPPID Sensors

antibody combination	binding subdomain on Axl	*K*_D_ (nM)^a^	LOD (pM)	maximal change in emission ratio
A-SB + B-LB	Ig2 + Ig2	270 + 19		
A-SB + C-LB	Ig2 + FN1	270 + 130	90	2.9-fold
A-SB + D-LB	Ig2 + FN2	270 + 1.09	63	3.7-fold
C-SB + B-LB	FN1 + Ig2	130 + 19	360	1.7-fold
D-SB + B-LB	FN2 + Ig2	1.09 + 19	21	6.6-fold
C-SB + D-LB	FN1 + FN2	130 + 1.09	8	9.6-fold

a *K*_D_ values of the antibodies used for the binding of sAxl as determined by SPR.

**Figure 2:**
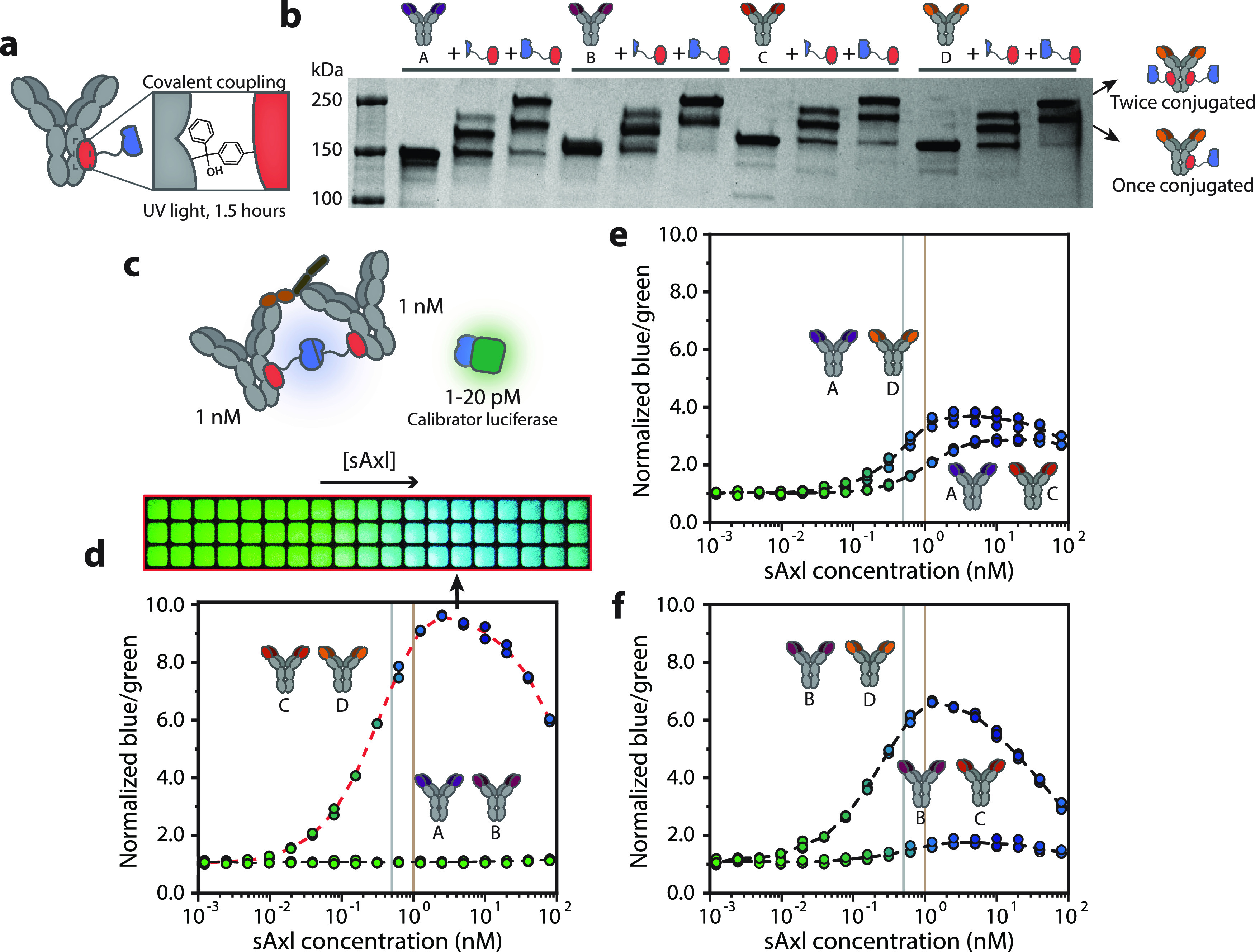
Development of sAxl-RAPPID assays. (a) SB or LB are covalently coupled to the four anti-Axl antibodies through protein G (Gx)-based photoconjugation. The photoreactive unnatural amino acid *p*-benzoylphenylalanine (*p*BpA) in Gx generates a covalent bond with the Fc-domain of the antibody upon irradiation with UV light (λ = 365 nm). (b) Nonreducing SDS-PAGE gel analysis of the photoconjugation of the four IgG1 anti-Axl antibodies to Gx-LB and Gx-SB. (c) Schematic overview of the sAxl-RAPPID, consisting of 1 nM antibody-SB, 1 nM antibody-LB, and 1–20 pM of calibrator luciferase. sAxl binding results in reconstituted NLuc and the increased emission of blue light. (d) Performance of the RAPPID assay consisting of C-SB with D-LB (with 20 pM calibrator luciferase), measured both with a plate reader and digital camera, and the dose–response curve of the RAPPID A-SB with B-LB monitored with a plate reader (with 1 pM calibrator luciferase). (e) Performance of antibody combinations A-SB with D-LB (with 7 pM calibrator luciferase) and A-SB with C-LB (with 5 pM calibrator luciferase). (f) Dose–response curves of D-SB with B-LB (with 24 pM calibrator luciferase) and C-SB with B-LB (with 2 pM calibrator luciferase). All luminescent assays were performed in PBS (pH 7.4, 0.1% (w/v) BSA) with 1500-fold diluted NLuc substrate. Blue-to-green ratios were calculated by dividing the emission at 458 nm by the emission at 518 nm. Gray and brown lines represent healthy sAxl concentration (∼0.5 nM) and sAxl concentrations associated with early HCC (∼1 nM), respectively. Individual data points (technical replicates, with *n* = 3 independent preparations of the analyte) are represented by circles, and dashed lines connect mean values.

To establish a panel of sAxl-RAPPID sensors, we used recombinant protein expression in *Escherichia coli* to obtain the sensor components Gx-LB and Gx-SB, composed of a large BiT (LB) or small BiT (SB, *K*_D_ = 2.5 μM) fragment of split NLuc fused to Gx.^31−33^ Upon illumination with UV light (λ = 365 nm), the unnatural amino acid *p*-benzoylphenylalanine (*p*BpA) in the protein G adaptor (Gx) forms a covalent bond with the Fc-domain of the anti-Axl antibody (Figure 2a). The four antibodies displayed efficient photoconjugation, as SDS-PAGE analysis showed conversion to primarily mono-conjugated and bi-conjugated species after mixing the antibody and sensor protein in a 1:4 molar ratio (Figure 2b). Subsequent to the production of the antibody-luciferase components, six different sensor combinations were analyzed for their performance to quantify sAxl. First, we scrutinized the performance of the two FNIII binding antibodies C and D, by adding increasing concentrations of sAxl to 1 nM of both sensor components and 20 pM of calibrator luciferase, followed by an incubation step of 1 h. This incubation step allowed for completed immunocomplex formation between antibody and analyte (Figure S4), enabling NLuc to reconstitute and resulting in increased blue light emission (Figure 2c). Light produced by an intensiometric assay is known to be susceptible to changes in environmental factors like pH and temperature and suffers from substrate depletion, decreasing the light intensity over time. These properties impede quantitative measurements and reduce the usability of the assay in a clinical or POC setting. Therefore, we introduced the green light-emitting calibrator luciferase, which enables ratiometric measurements by comparing the blue light emitted by the complemented split NLuc of the sensor with the green light of the calibrator (Figure S4).^32^ The required amount of calibrator luciferase depends on the absolute luminescent signal that is produced in a specific assay and should be optimized accordingly. The FNIII binding CD-RAPPID assay, composed of antibody C conjugated to Gx-SB and antibody D fused to Gx-LB, displayed an sAxl concentration-dependent change in color from green to blue, with a low limit of detection of 8 pM (Figure 2d). This response could be detected both by plate reader analysis and in images recorded with a simple digital camera. The blue-to-green ratio decreased again at sAxl concentrations exceeding 3 nM due to the “hook” effect, which occurs when the sensor components bind to distinct sAxl proteins, prohibiting the complementation of NLuc. As expected, AB-RAPPID, comprising the two antibodies binding to the Ig-like repeats of Axl, did not show any increase in blue luminescence (Figure 2d). This lack of response is caused by the overlapping binding sites of the antibodies, impeding the binding of two sensor components to the same sAxl protein.^22^ We also tested several combinations of FNIII- and Ig-binders. All four sensors (AD-RAPPID, AC-RAPPID, BD-RAPPID, and BC-RAPPID) displayed an increase in blue-to-green ratio upon increasing concentrations of sAxl and exhibited LODs of 63, 90, 21, and 360 pM, respectively (Figure 2e,f and Table 1). The RAPPID assays containing the high-affinity antibody D showed higher relative responses and lower LODs, demonstrating the importance of having at least one high-affinity antibody in the sensor format. Nevertheless, the CD-RAPPID assay, combining antibodies targeting the FN1 and FN2 domains, exhibited the highest maximal change in emission ratio, suggesting that adjacent but nonoverlapping binding sites contribute to more efficient NLuc complementation. We therefore chose CD-RAPPID for subsequent measurements in blood plasma.^37,38^

### sAxl Detection in Human Blood Plasma

**Figure 3:**
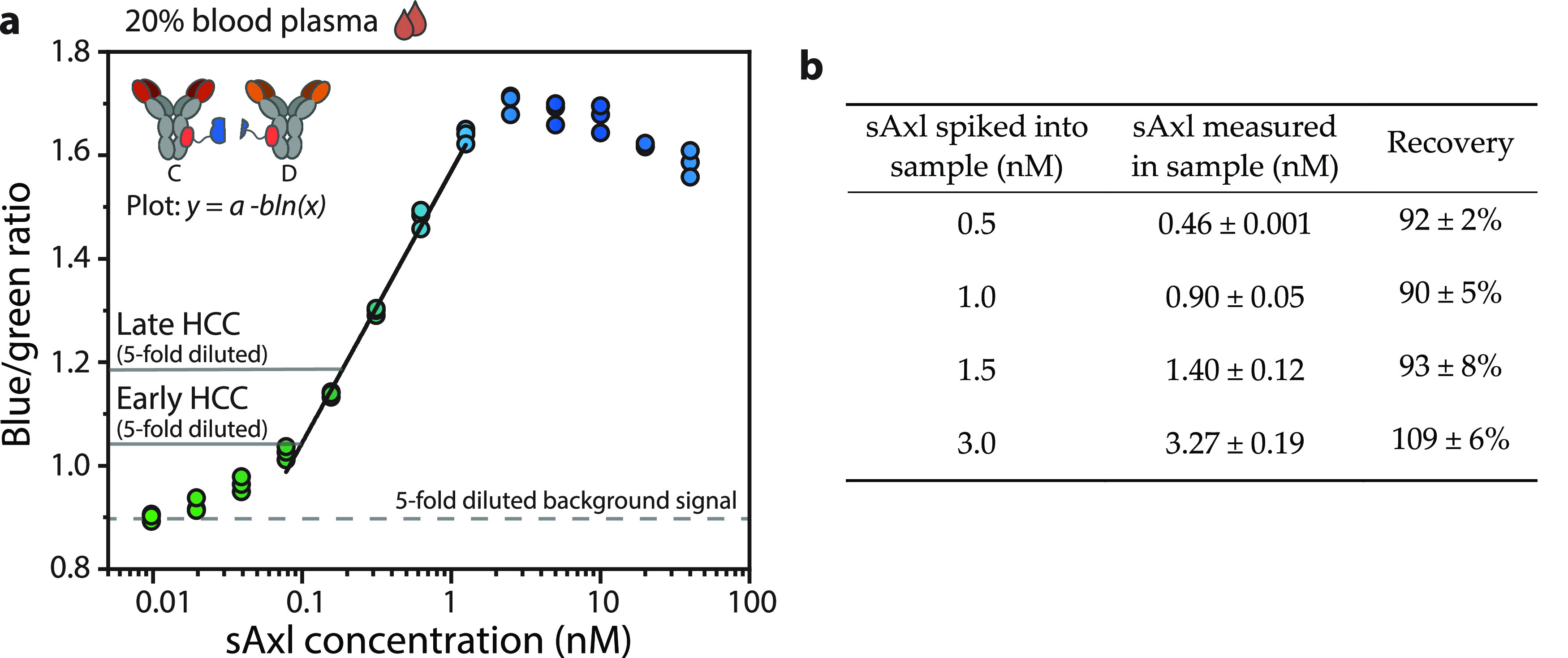
Detection of blood plasma sAxl levels associated with early and late HCC. (a) Calibration curve in 20% plasma, diluted with PBS (pH 7.4, 0.1% (w/v) BSA). A final concentration of 1 nM C-SB, 1 nM D-LB, 8 pM calibrator luciferase, and 400× diluted NLuc substrate was added. Concentrations related to early HCC and late HCC are presented as a, respectively, 0.5 nM increase and 0.93 nM increase and corrected for 5-fold dilution (0.1 and 0.186 nM increase, respectively). The black line represents a linear curve that was fit through the linear part of the data. Individual data points (technical replicates, with *n* = 3 independent preparations of the analyte) are represented by circles. (b) Comparison between the known spiked concentration of sAxl and the concentration measured with CD-RAPPID, by making use of the blue-to-green ratios of the calibration curve in (a). The curve corresponding to the data can be found in Figure S5. Data in the table represent mean values ± s.d. from technical replicates, with *n* = 3 independent preparations of the analyte.

To demonstrate the potential of the CD-RAPPID sensor for diagnosing HCC, we employed the sensor to measure therapeutically relevant concentrations of sAxl in human blood plasma. The high sensitivity of the RAPPID sensor allowed for 5-fold sample dilution, reducing potential matrix effects such as absorption of blue light by biliverdin.^32,37^ Using 1 nM of both sensor components and 8 pM calibrator luciferase, increasing amounts of sAxl were added to 20% human blood plasma and a dose–response curve with a two-fold maximal change in emission ratio was observed (Figure 3a). Please note that in this case the plasma already contained approximately 0.5 nM sAxl, resulting in a background of 0.1 nM sAxl in the final assay and reducing the maximal change in emission ratio. The sensor was responsive in the physiologically relevant low nanomolar concentration range and exhibited a LOD, after correcting for the 5-fold dilution, of 73 pM in 20% plasma. The additional 0.1 and 0.186 nM increase in sAxl concentration, corresponding to the 1 and 1.43 nM plasma concentrations of Axl in early and late HCC, respectively, could clearly be distinguished from the background level of sAxl present in normal plasma. Next, we spiked known concentrations of sAxl in human blood plasma and applied the RAPPID sensor to measure the corresponding blue-to-green ratios. Accordingly, four different sAxl concentrations (0.5, 1.0, 1.5, and 3.0 nM) were added to 4 μL plasma samples and diluted 5-fold in the sensor mixture. The calibration curve in Figure 3a was subsequently used to translate the blue-to-green ratios measured in the spiked plasma samples to sAxl concentrations. After correction for the five-fold dilution, we compared the results obtained with the RAPPID sensor to the known spiked sAxl concentrations and found a good correlation (Pearson’s *r* = 0.994) and a recovery between 92 ± 2 and 109 ± 6% (Figures S5 and 3b, respectively). Together, these results show that the intrinsic ratiometric detection of the sensor results in excellent reproducibility, enabling the reliable detection of small differences in both target analyte concentrations and emission ratios.

### Detection of Shedded sAxl in Cell Culture Medium

**Figure 4:**
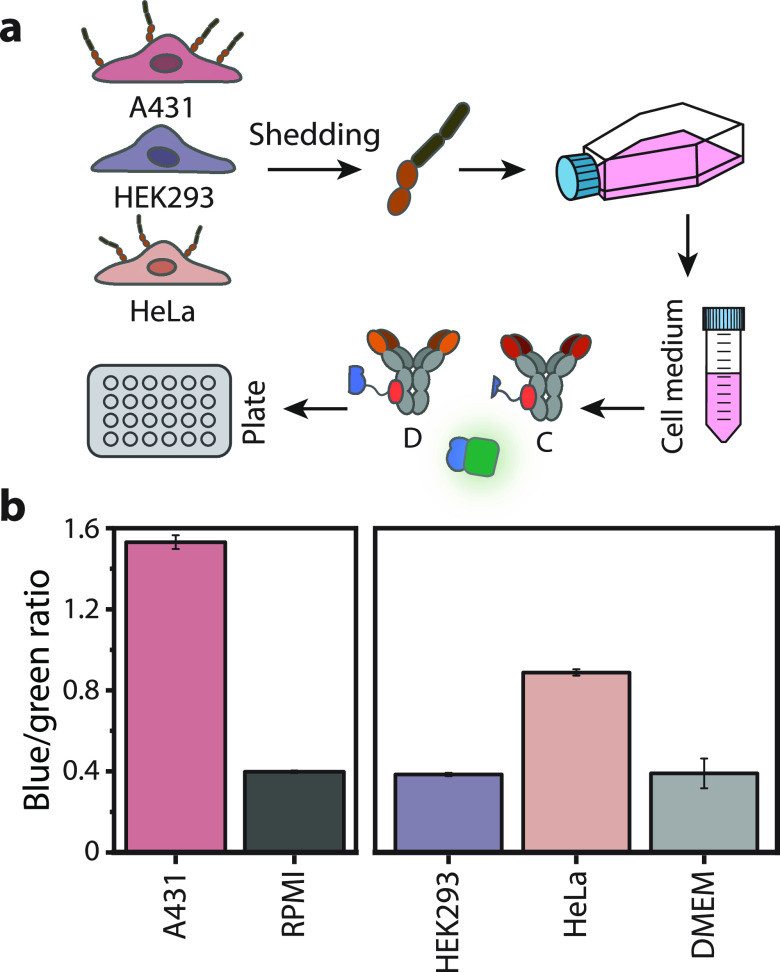
Detection of shedded Axl in cell culture medium. (a) Schematic representation of the CD-RAPPID assay for the detection of sAxl in medium of Axl-expressing cell lines. Medium from A431 (RPMI), HeLa and Hek293 cells (DMEM) were collected and diluted with PBS (pH 7.4, 0.1% (w/v) BSA) and sensor mixture (1 nM D-LB, 1 nM C-SB and 2 pM calibrator luciferase) to a final medium concentration of 10%. Before the addition of 1500-fold diluted NLuc substrate, the mixture was incubated for 1 h at room temperature. (b) Ratiometric detection of sAxl in cell medium collected from A431, HeLa, and HEK293 cells. Ratios for DMEM and RPMI represent controls of fresh culture medium. Bars in the histogram represent mean values ± s.d. from technical replicates, with *n* = 3.

sAxl can appear in blood plasma as a result of proteolytic cleavage of cell surface Axl by metalloproteinases ADAM10 and ADAM17.^39,40^ Shedding of the ectodomain of Axl and the subsequent elevated release of sAxl have been associated with several other types of cancer, including renal carcinoma^41^ and melanoma.^42^ To investigate if RAPPID can be used to measure shedding of cell surface Axl, we applied the CD-RAPPID sensor to measure the presence of sAxl in the medium of Axl-expressing cell lines. The culture medium from two Axl-expressing cancer cell lines (A431 and HeLa) and one Axl-negative control cell line (HEK293) were collected. Following 1 h incubation with 0.6 nM sensor mixture and 2 pM calibrator luciferase, substrate was added and the ratio of blue and green luminescence was measured (Figure 4a). Medium collected from A431 and HeLa cells displayed an increased blue-to-green ratio compared to fresh RPMI and DMEM culture medium controls, implying the presence of shedded Axl (Figure 4b). As expected, the cell medium harvested from the HEK293 cells did not display this increase in blue-to-green ratio after incubation with the RAPPID sensor mixture, confirming the absence of Axl shedding. These results illustrate that the RAPPID sensor provides an attractive tool to monitor receptor shedding and identify cells that are subjected to this type of proteolytic cleavage.

### RAPPID for the Detection of Cell Surface Receptors

**Figure 5:**
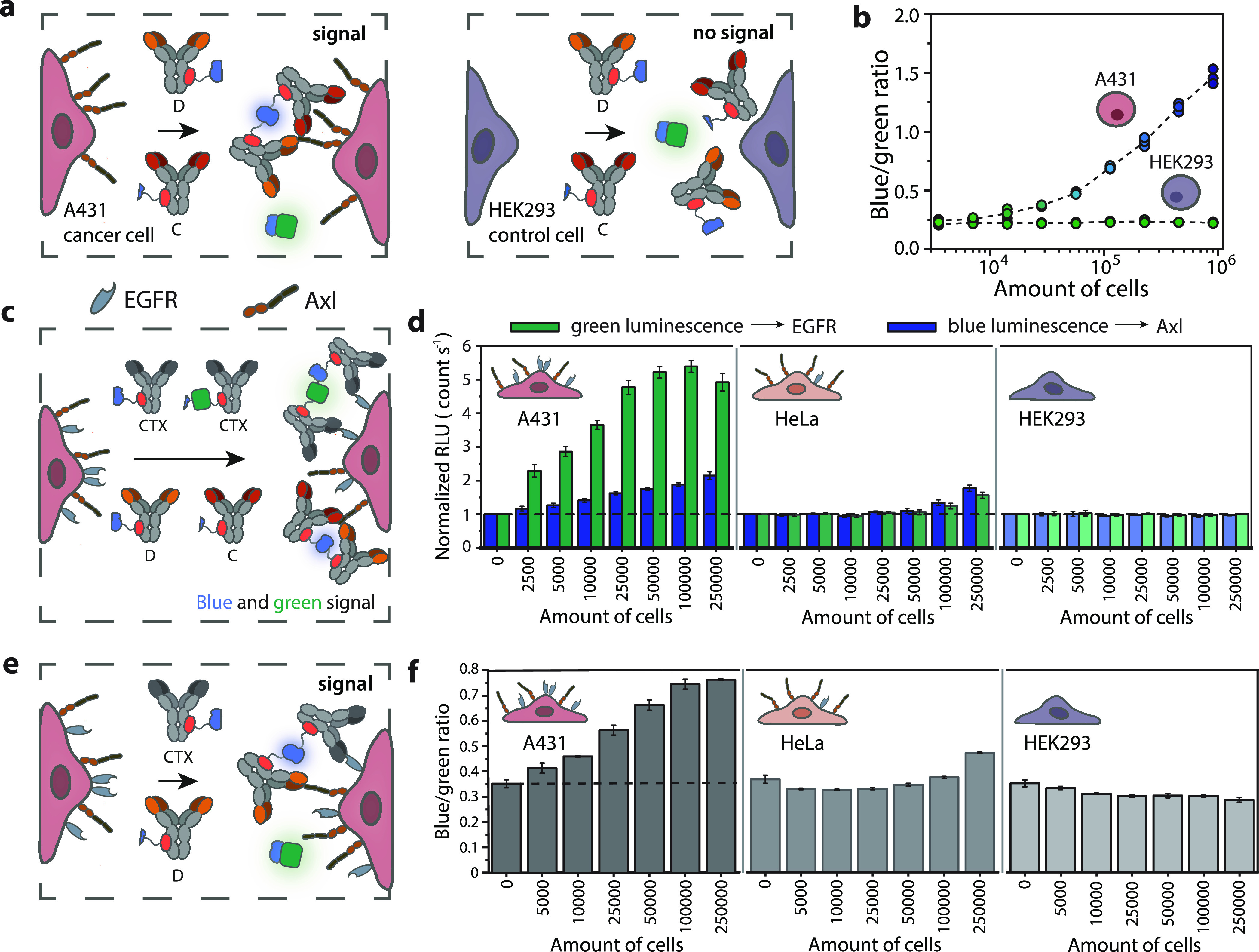
RAPPID assay for the detection of cell surface receptors. (a) Axl-expressing A431 cancer cells and HEK293 control cells were released from the culture flask using trypsin and subsequently incubated with 10 pM calibrator luciferase, D-LB, and C-SB (0.75 nM each) to allow complex formation with the cell surface receptor Axl. (b) Response curves of CD-RAPPID to an increasing number of A431 or HEK293 cells. Individual data points (technical replicates, with *n* = 3 independent dilutions of the cells) are represented by circles, and dashed lines connect mean values. (c) CTX-mNG-SB, CTX-LB, D-LB, and C-SB (0.25 nM of the EGFR RAPPID and 0.6 nM of the Axl RAPPID) are incubated with A431, HeLa, and HEK293 cells. The presence of EGFR on the cellular membrane induces the emission of green light, while Axl binding produces a blue light signal. (d) Luminescent response to increasing amounts of cells. Blue (λ = 458 nm) and green (λ = 518 nm) bioluminescent signals were monitored on a plate reader and normalized for background light emission. (e) AND-gate RAPPID, with 0.6 nM CTX-LB, 0.6 nM D-SB and 5 pM calibrator luciferase, producing light only in the presence of both EGFR and Axl. (f) Sensor response to an increase in A431, HEK293, or HeLa cells. All experiments were executed in PBS (pH 7.4, 0.1% (w/v) BSA). Bars in the histograms represent mean values ± s.d., from technical replicates, with *n* = 3 independent dilutions of the cells.

Cell surface receptors such as Axl and the epidermal growth factor receptor (EGFR) are overexpressed in several types of cancers and therapeutic treatments that target these receptors are currently used in clinical care.^43−49^ Therefore, the detection of these receptors is important for treatment decision making and informs on disease progression. RAPPID could provide an easy and cheap alternative for currently used analysis methods such as FACS, which requires access to advanced flow cytometry equipment. Accordingly, we analyzed whether RAPPID allows distinction of different tumor cell lines based on the overexpression of Axl and EGFR. To allow the detection of cell surface Axl, we first dissociated adhesive Axl-expressing A431 cancer cells or HEK293 control cells and washed the cells with PBS to prevent the detection of shedded Axl. Subsequently, the sensor mixture, consisting of CD-RAPPID and 10 pM of calibrator luciferase, was incubated with an increasing amount of either A431 or HEK293 cells (Figure 5a). When Axl is displayed on the membrane of cells, the antibody-luciferase conjugates can bind and subsequently emit blue light as a result of NLuc reconstitution. Figure 5b shows that increasing the number of A431 cells induced a higher blue-to-green ratio, suggesting an elevated amount of Axl in the mixture. Increasing the amount of HEK293 cells did not cause a change in emission ratio, confirming the absence of cell surface Axl.

To identify two different cell surface receptors in parallel, we next developed a new, green variant of RAPPID by introducing the green fluorescent protein mNeonGreen (mNG) in the Gx-SB construct, generating Gx-mNG-SB (Figure S2). Binding of antibody-conjugated Gx-LB and Gx-mNG-SB to the target analyte induces bioluminescence resonance energy transfer (BRET) between the restored split NLuc and mNG, generating a green bioluminescent output signal (Figure S6). We applied this dual-color platform to allow simultaneous detection of Axl and EGFR. EGFR is known to be able to form ligand-independent homodimers, enabling the adjacent binding of one antibody type to two distinct EGFR proteins and the subsequent complementation of NLuc.^50−54^ Accordingly, we photoconjugated Gx-mNG-SB and Gx-LB to the EGFR-binding therapeutic antibody cetuximab (CTX, Figure S7) and Gx-LB and Gx-SB to anti-Axl D and C, respectively. After incubating the EGFR-green and Axl-blue sensor components with A431, Hela, and HEK293 cells, the blue (458 nm) and green (518 nm) light output, corresponding to Axl and EGFR binding, respectively, were measured using a plate reader (Figure 5c). An elevated blue and green light signal was observed when increasing the amount of A431 cells, consistent with the presence of both Axl and EGFR (Figure 5d). The dual-color RAPPID assay with HeLa cells also showed an increase in blue and green light, suggesting the presence of both Axl and EGFR. However, HeLa cells display a lower EGFR and Axl density compared to A431 cells (Figures S6 and S8, respectively).^55^ Therefore, the response of the sensors with the HeLa cells is smaller than with A431 cells and only occurs in the presence of a large amount of cells. As expected, the HEK293 cells did not display a change in either green or blue light when increasing the number of cells, confirming the absence of both EGFR and Axl (Figures S6 and S8, respectively). Very similar results were obtained when the green RAPPID was used for Axl detection and the blue RAPPID for EGFR detection, demonstrating the ease of exchanging readout modules (Figures S9 and S10).

Finally, we explored whether a RAPPID sensor could be developed that would only respond when both Axl and EGFR are expressed on the same cell, thus representing a bioluminescent “AND-gate” (Figure 5e). We photoconjugated CTX and anti-Axl D to, respectively, Gx-LB and Gx-SB, and added an increasing amount of A431, HeLa, or HEK293 cells in the presence of 5 pM calibrator luciferase. The A431 cells, overexpressing both Axl and EGFR, display an increase in blue-to-green ratio (Figure 5f). HeLa cells have a lower EGFR and Axl expression compared to A431 cells (Figures S6 and S8, respectively),^55^ resulting in a relatively small increase in blue-to-green ratio. HEK293 cells lack both required input receptors, precluding the reconstitution of split NLuc and the corresponding increase in blue-to-green ratio. Previous research has suggested that Axl and EGFR can form heterodimers and that this interaction is associated with EGFR drug resistance in several types of cancers.^56−61^ Our data support that EGFR and Axl are in very close proximity on the cellular membrane of A431 cells, as anti-Axl antibody D and CTX, enabled by the semiflexible linkers that can span 10–15 nm, can bind adjacent sites and produce blue light in the presence of both input receptors. Collectively, these results illustrate that RAPPID can be employed to detect Axl-expressing cells and that the platform can be easily adapted to identify cells that display two different cell surface receptors using dual-color RAPPID or AND-gate RAPPID.

## Conclusions

Herein, we developed six RAPPID sensors and screened them for their ability to discriminate between healthy sAxl levels and concentrations associated with early HCC. The best-performing RAPPID sensor, with a picomolar limit of detection and a >9-fold maximal change in emission ratio, was subsequently applied to successfully detect clinically relevant sAxl concentrations in spiked blood plasma. In addition to measuring (patho)physiological sAxl concentrations, the RAPPID sensors can also be applied to identify cell lines that experience receptor shedding and detect the concurrent presence of two cell surface proteins. The broad scope of applications makes the RAPPID sensors attractive tools for both point-of-care diagnostic purposes and in clinical decision making. This can be envisioned by integrating the sAxl-RAPPID sensors in low-cost microfluidic point-of-care cartridges or paper-based devices. Furthermore, provided that suitable antibodies are available, the newly developed green RAPPID assay allows the sensor platform to be extended to measure other HCC-related biomarkers such as α-fetoprotein. The dual-color multiplex detection of two biomarkers could further increase sensitivity and specificity.

The homogeneous nature and intrinsic low background signal of the RAPPID sensors eliminate the need for the washing steps currently used in immunostaining. Additionally, the semiflexible linker of the sensors can span distances between two domains within single protein targets and even between different cell surface receptors. Hence, the RAPPID sensors show potential for the fast and user-friendly multiplexed detection of membrane biomarkers and might be utilized to measure cancer-related cell surface receptors in tissue sections. Furthermore, the bioluminescent signal of the sensors can be distinguished with a plate reader or a simple digital camera, making the RAPPID platform an attractive substitute to expensive and relatively complex cellular detection techniques like FACS. In the future, this sensor platform can be extended to allow measuring three membrane receptors simultaneously. Accordingly, a triple-color readout system could be established by developing a red-shifted RAPPID variant. Alternatively, the AND-gate RAPPID could be expanded by introducing a tri-part system, comprising two smaller and one larger NLuc fragment.^62,63^ With these systems, other therapeutically relevant receptors like HER2 could be included in the cellular screening process.^64,65^ Furthermore, the current dual-color RAPPID prohibits the employment of the green calibrator luciferase. Therefore, a red-shifted calibrator luciferase could be introduced, enabling also robust ratiometric measurements in multiplex assays.

## Supporting Information Available

DNA sequence of Gx-mNG-SB, SDS-PAGE gel analyses of protein purification and photoconjugation, SPR data of anti-Axl antibodies, and luminescent spectra of the green and blue RAPPID (PDF)
