# Supplementary material for: Bioluminescent RAPPID Sensors for the Single-Step Detection of Soluble Axl and Multiplex Analysis of Cell Surface Cancer Biomarkers
Source: Anal Chem. 2022 Apr 19;94(17):6548–56. doi: 10.1021/acs.analchem.2c00297 (PMC9069438; doi:10.1021/acs.analchem.2c00297)
Supplement: Supplementary file 1 — ac2c00297_si_001.pdf [file ac2c00297_si_001.pdf]

Supporting information

Bioluminescent RAPPID sensors for the single-step detection of soluble Axl and multiplex analysis of cell surface cancer biomarkers

Eva A. van Aalen[ab], Simone F. A. Wouters[ab†], Dennis Verzijl[c] and Maarten Merkx*[ab]

[a] Laboratory of Chemical Biology, Department of Biomedical Engineering, Eindhoven University of Technology, P.O Box 513, 5600 MB Eindhoven, The Netherlands.
[b] Institute for Complex Molecular Systems, Eindhoven University of Technology, P.O Box 513, 5600 MB Eindhoven, The Netherlands.
[c] Genmab, 3584 CT, Utrecht, The Netherlands.

Content **Page**

Figure S1	DNA and amino acid sequence of Gx-mNG-SB	S2
Figure S2	Expression and purification of Gx-mNG-SB	S3
Figure S3	Binding affinities of the four anti-Axl antibodies	S3
Figure S4	Intensiometric and ratiometric bioluminescent detection with CD-RAPPID	S4
Figure S5	Human blood plasma samples spiked with sAxl	S4
Figure S6	Green EGFR RAPPID with A431, HeLa or HEK293 cells	S5
Figure S7	Photoconjugation of Gx-mNG-SB and Gx-LB to Cetuximab (CTX)	S6
Figure S8	Blue Axl RAPPID with A431, HeLa or HEK293 cells	S7
Figure S9	Photoconjugation of green Axl RAPPID and blue EGFR RAPPID	S8
Figure S10	Performance of the green Axl RAPPID and blue EGFR RAPPID	S9

```
M  G  W  S  H  P  Q  F  E  K  G  G  S  M  T  F  K  L  I  I
ATGGGCTGGAGCCATCCGCAGTTTGAAAAAGGTGGTAGCATGACATTTAAACTGATTATC

N  G  K  T  L  K  G  E  I  T  I  E  A  V  D  A  *  E  A  E
AACGGCAAAACTTTAAAGGGAGAGATCACAATAGAAGCGGTGGATGCTTAGGAGGCGGAG

K  I  F  K  Q  Y  A  N  D  Y  G  I  D  G  E  W  T  Y  D  D
AAGATTTTTAAGCAGTATGCAAATGATTATGGAATTGATGGTGAATGGACTTATGACGAC

A  T  K  T  F  T  V  T  E  E  F  T  G  G  S  G  G  S  G  G
GCAACTAAAACTTTCACGGTAACAGAAGAATTTACAGGAGGTTCGGGTGGGTCGGGAGGT

S  G  G  S  G  G  S  G  G  S  G  E  F  A  E  A  A  A  K  E
TCTGGCGGCTCTGGAGGAAGTGGTGGTAGCGGTGAATTCGCCGAAGCAGCCGCTAAAGAA

A  A  A  K  E  A  A  A  K  E  A  A  A  K  E  A  A  A  K  E
GCCGCAGCAAAGGAAGCCGCGGCCAAGGAGGCAGCCGCAAAAGAGGCCGCGGCGAAGGAA

A  A  A  K  A  E  F  G  G  S  G  G  S  G  G  S  G  G  S  G
GCAGCAGCCAAGGCAGAATTCGGGGGTAGCGGCGGCTCGGGGGGTAGTGGTGGAAGCGGG

G  S  G  T  S  G  G  H  M  V  S  K  G  E  E  D  N  M  A  S
GGTTCAGGCACTAGTGGTGGTCATATGGTAAGTAAAGGTGAAGAAGACAATATGGCTTCT

L  P  A  T  H  E  L  H  I  F  G  S  I  N  G  V  D  F  D  M
CTGCCTGCCACACATGAGCTTCATATTTTTGGGAGCATAAACGGAGTGGATTTCGACATG

V  G  Q  G  T  G  N  P  N  D  G  Y  E  E  L  N  L  K  S  T
GTAGGTCAGGGTACGGGGAACCCTAACGATGGATATGAGGAGTTGAATCTTAAAAGCACA

K  G  D  L  Q  F  S  P  W  I  L  V  P  H  I  G  Y  G  F  H
AAGGGTGATCTGCAGTTCTCGCCCTGGATCCTGGTGCCGCATATAGGTTATGGTTTCCAT

Q  Y  L  P  Y  P  D  G  M  S  P  F  Q  A  A  M  V  D  G  S
CAGTATCTTCCATACCCGGATGGCATGAGCCCTTTTCAGGCCGCAATGGTAGATGGCTCA

G  Y  Q  V  H  R  T  M  Q  F  E  D  G  A  S  L  T  V  N  Y
GGATATCAAGTGCATCGGACCATGCAGTTTGAAGATGGGGCGTCTTTGACGGTAAATTAC

R  Y  T  Y  E  G  S  H  I  K  G  E  A  Q  V  K  G  T  G  F
AGGTACACCTATGAGGGTAGCCATATAAAGGGAGAAGCGCAGGTGAAGGGAACTGGATTC

P  A  D  G  P  V  M  T  N  S  L  T  A  A  D  W  C  R  S  K
CCAGCGGATGGCCCAGTCATGACAAACAGCCTCACCGCTGCTGATTGGTGCCGATCCAAG

K  T  Y  P  N  D  K  T  I  I  S  T  F  K  W  S  Y  T  T  G
AAAACGTATCCAAACGATAAAACTATCATTTCTACTTTTAAGTGGTCCTATACAACAGGA

N  G  K  R  Y  R  S  T  A  R  T  T  Y  T  F  A  K  P  M  A
AACGGGAAACGCTATCGTTCAACGGCCCGCACGACCTACACGTTTGCAAAGCCAATGGCT

A  N  Y  L  K  N  Q  P  M  Y  V  F  R  K  T  E  L  K  H  S
GCGAATTATCTGAAAAACCAGCCGATGTATGTGTTCCGTAAAACCGAACTGAAACATTCT

K  T  E  L  N  F  K  E  W  Q  K  A  F  T  G  S  G  G  T  V
AAAACGGAGCTCAATTTCAAGGAATGGCAGAAGGCATTTACGGGTAGCGGTGGTACCGTT

T  G  Y  R  L  F  E  K  E  S  G  G  S  H  H  H  H  H  H  *
ACCGGCTATCGTCTGTTTGAAAAAGAGAGCGGCGGTTCACATCATCATCACCACCATTAA
```

Figure S1. DNA and amino acid sequence of Gx-mNG-SB. Strep-tag (gray), protein G domain (red), amber stop codon (yellow), mNG (green), SB (blue) His-tag (pink).

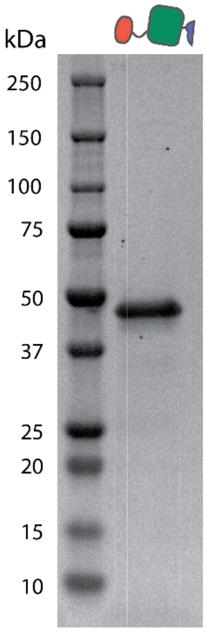

Figure S2. Expression and purification of Gx-mNG-SB. Reducing SDS-PAGE (4-20%) analysis of Gx-mNG-SB. After expression of the fusion protein in *E. coli*, Gx-mNG-SB was purified using Ni^{2+} affinity chromatography and Strep-Tactin chromatography.

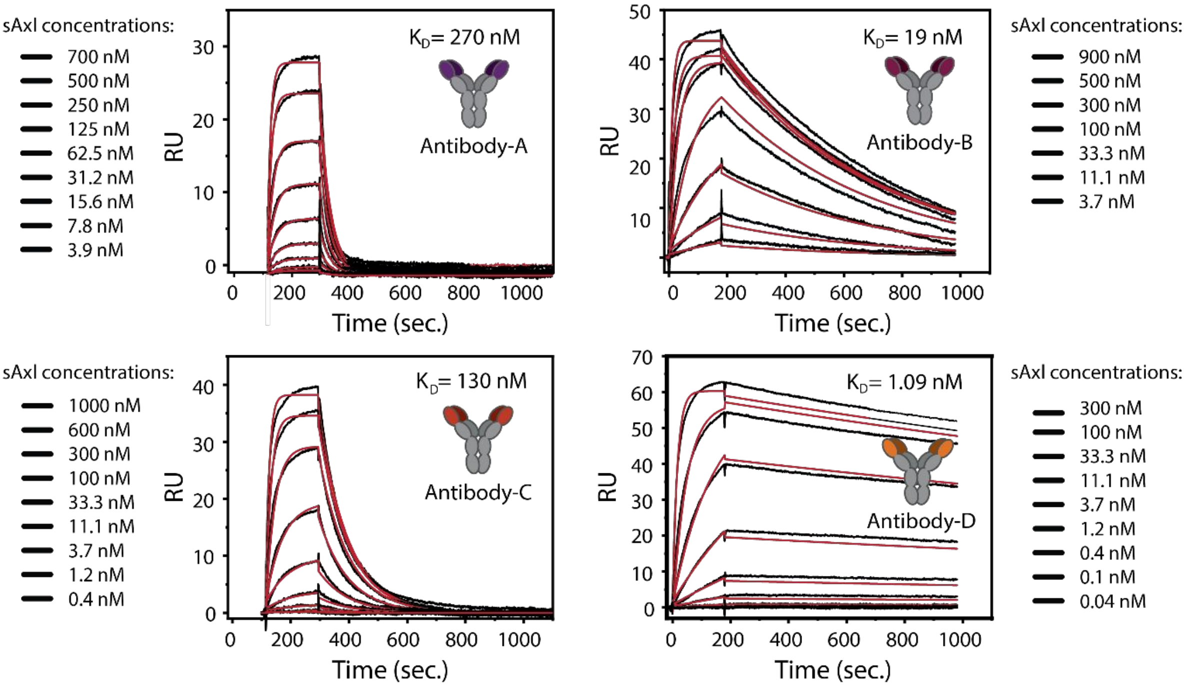

Figure S3. Binding affinities of the four anti-Axl. Surface plasmon resonance (SPR) sensorgrams are shown in black and fitted curves (kinetics 1:1 binding model fit) in red. Anti-Axl antibodies (A, B, C and D) were immobilized on protein G chips and different concentrations of sAxl were flown over the surface in HBS-EP buffer (10 mM HEPES, 150 mM NaCl, 3 mM EDTA, 0.05% v/v P20, pH7.4). SPR measurements were executed on a Biacore X100.

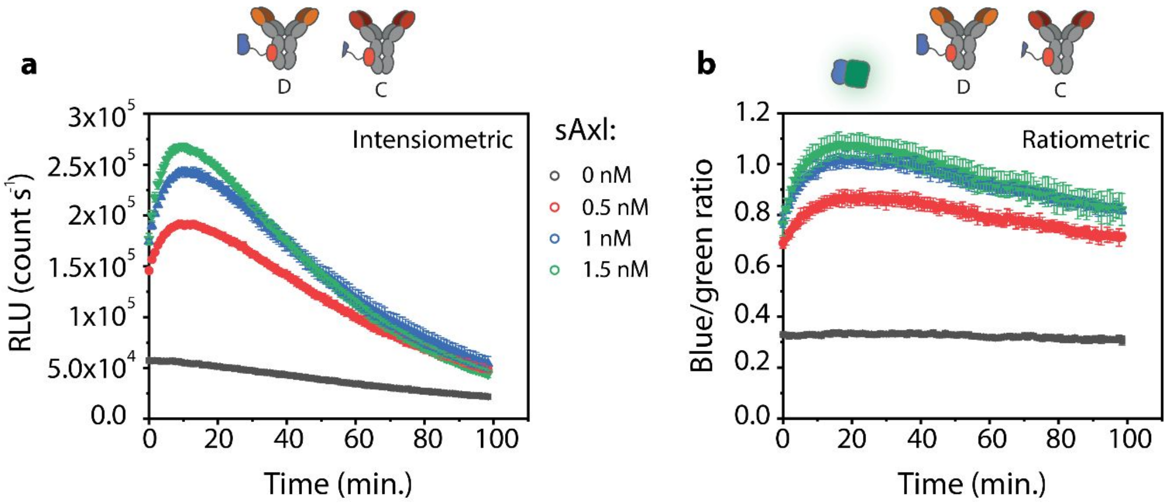

Figure S4. Intensiometric and ratiometric bioluminescent detection with CD-RAPPID. (a) Performance of the intensiometric CD-RAPPID (without calibrator luciferase). (b) Performance of the ratiometric CD-RAPPID (with 20 pM calibrator luciferase). For both assays, 1500-fold diluted NLuc substrate was added to 1 nM of D-LB and 1 nM of C-SB. All components (sensor, calibrator, analyte and substrate) were added simultaneously at t=0 and bioluminescence was directly monitored for ~100 min at 20 °C.

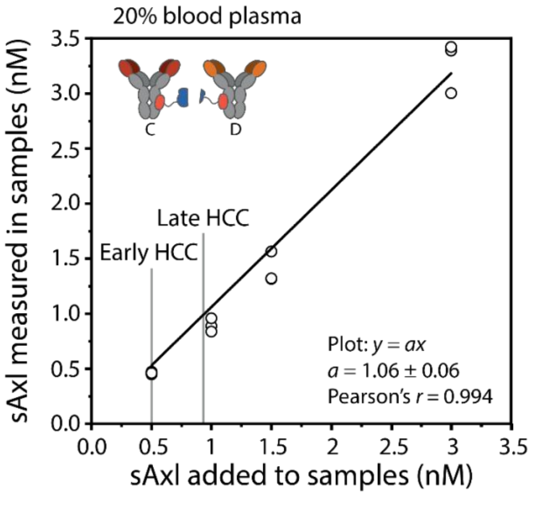

Figure S5. Human blood plasma samples spiked with sAxl. Comparison between the known spiked concentration of sAxl (x-axis) and the concentration measured with CD-RAPPID (y-axis), by making use of the blue/green ratios of the calibration curve in Figure 3a. Found concentrations are multiplied by 5 to correct for sample dilution. Individual data points (technical replicates, with n = 3 independent dilutions of sAxl in plasma) are represented as circles and line indicates a linear fit, with a slope of 1.06 ± 0.06 and Pearson's r of 0.994.

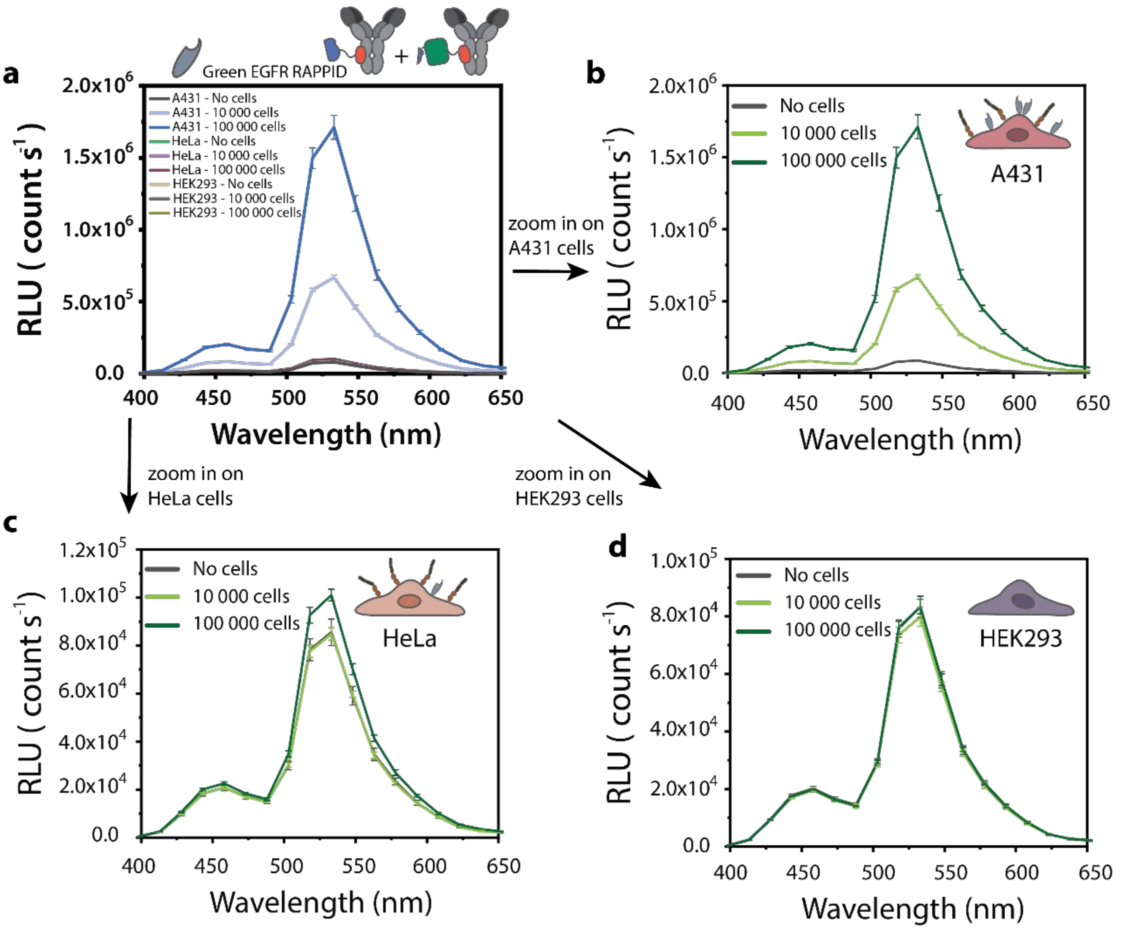

Figure S6. Green EGFR RAPPID with A431, HeLa or HEK293 cells. (a) Bioluminescent response of the green EGFR RAPPID, consisting of 0.6 nM CTX-LB and 0.6 nM CTX-mNG-SB, to the addition of 10 000 or 100 000 A431, HeLa or HEK293 cells. (b) Zoom in of the A431 data presented in panel a. (c) Zoom in of the data corresponding to the HeLa cells in panel a. (d) Zoom in of the HEK293 data shown in panel a. All experiments were executed in PBS buffer (pH 7.4, 0.1% (w/v) BSA) with 1500-fold diluted NanoLuc substrate. Lines represent mean values ± s.d., from technical replicates, with n = 3 independent dilutions of the cells.

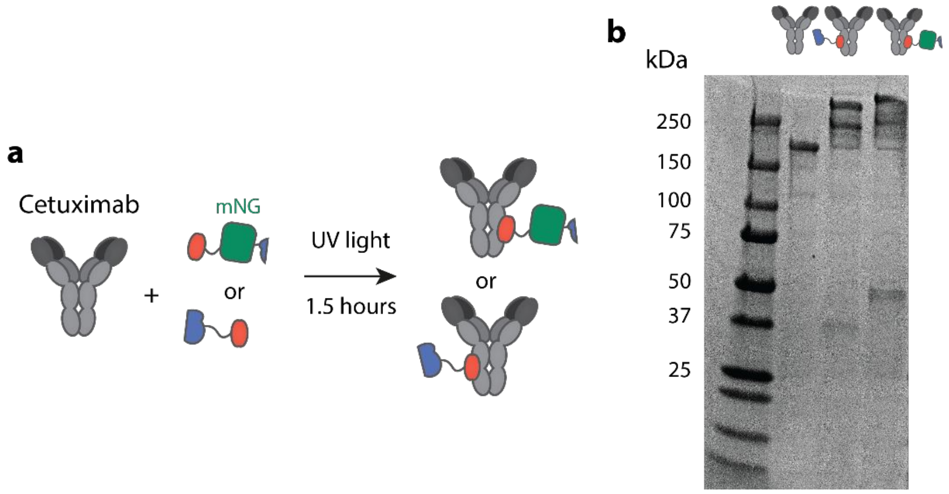

Figure S7. Photoconjugation of Gx-mNG-SB and Gx-LB to Cetuximab (CTX). (a) Photoconjugation was performed with 1 µM CTX and 4 µM Gx-mNG-SB or Gx-LB in PBS buffer (pH 7.4) for 1.5 hours. (b) Non-reducing SDS-PAGE analysis of the CTX conjugation, displaying non-conjugated, single- and twice-conjugated products.

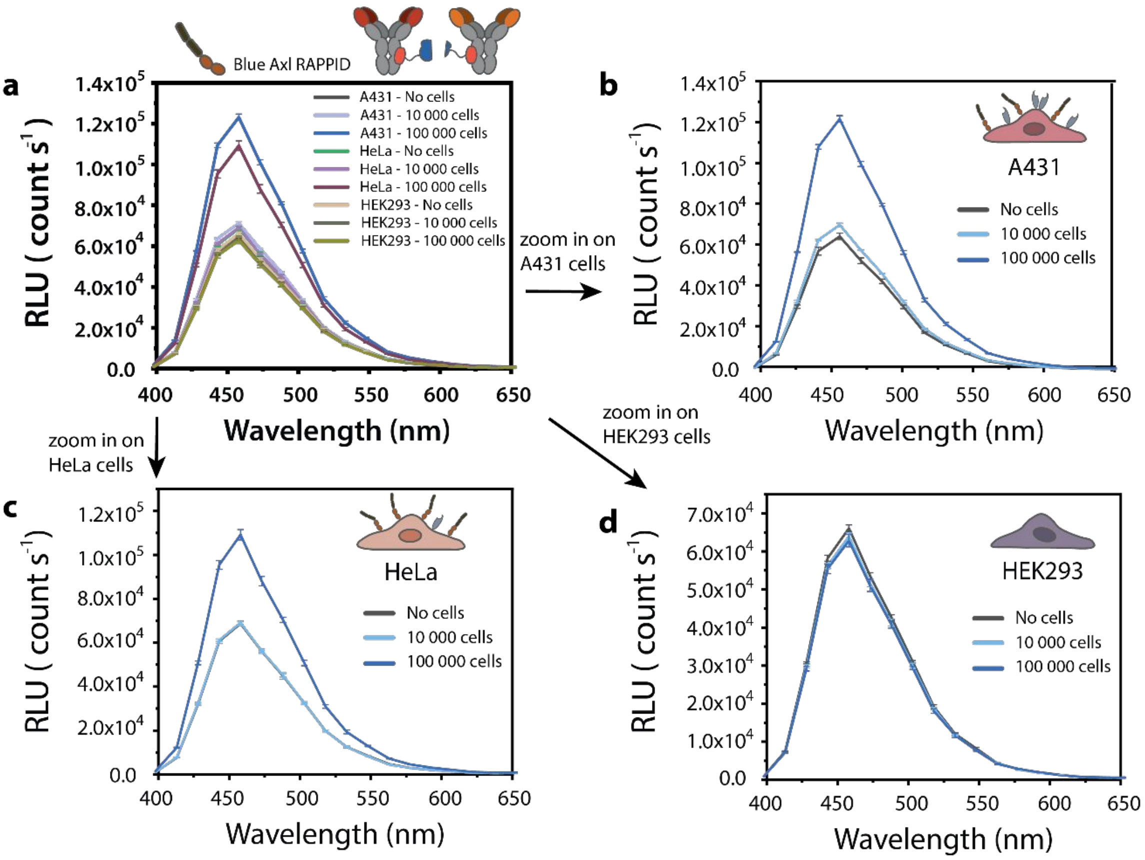

Figure S8. Blue Axl RAPPID with A431, HeLa or HEK293 cells. (a) Bioluminescent response of the blue Axl RAPPID, consisting of 0.6 nM D-LB and 0.6 nM C-SB, to the addition of 10 000 or 100 000 A431, HeLa or HEK293 cells. (b) Zoom in of the A431 data presented in panel a. (c) Zoom in of the data corresponding to the HeLa cells in panel a. (d) Zoom in of the HEK293 data shown in panel a. All experiments were executed in PBS buffer (pH 7.4, 0.1% (w/v) BSA) with 1500-fold diluted NanoLuc substrate. Lines represent mean values ± s.d., from technical replicates, with n = 3 independent dilutions of the cells.

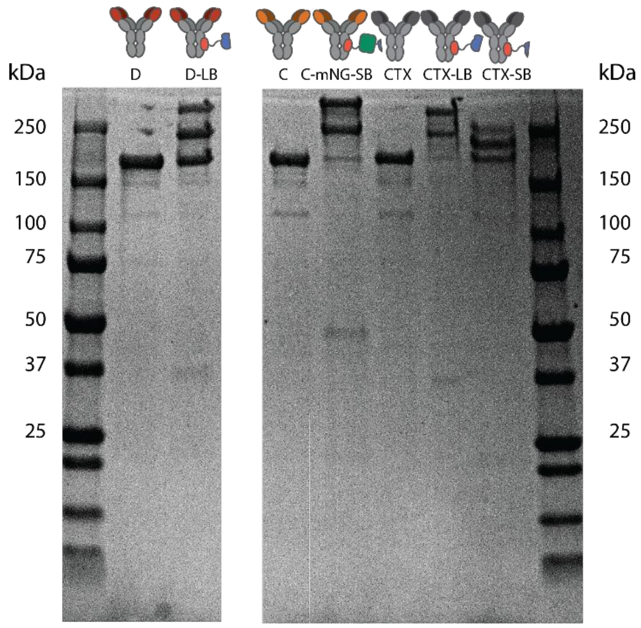

Figure S9. Photoconjugation of green Axl RAPPID and blue EGFR RAPPID. Non-reducing SDS-PAGE (4-20%) analysis of anti-Axl D conjugated to Gx-LB, anti-Axl C to Gx-mNG-SB and CTX to both Gx-LB and Gx-SB. Photoconjugation was performed with 1 µM antibody and 4 µM sensor protein in PBS buffer (pH 7.4) for 1.5 hours.

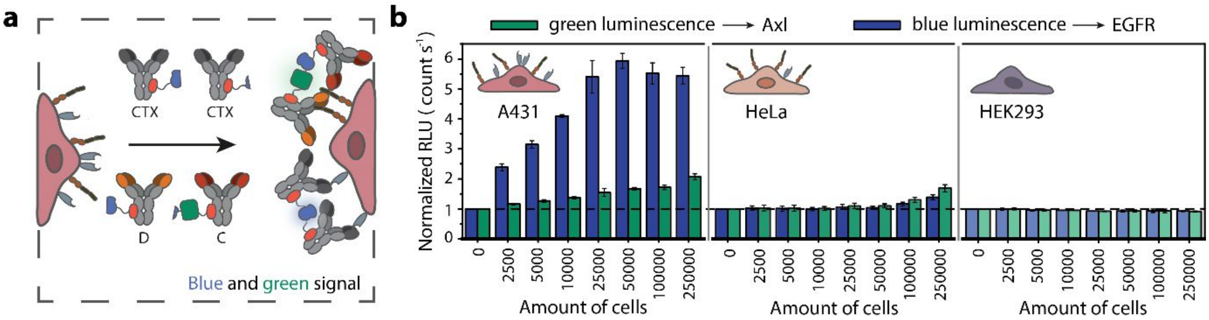

Figure S10. Performance of the green Axl RAPPID and blue EGFR RAPPID. (a) Green Axl RAPPID (0.6 nM), consisting of C-mNG-SB and D-LB, and blue EGFR RAPPID (0.6 nM), with CTX-SB and CTX-LB, were incubated with either A431, HeLa or HEK293 cells. (b) Normalized response curves of the green Axl and blue EGFR RAPPID to increasing amounts of cells. All experiments were executed in PBS buffer (pH 7.4, 0.1% (w/v) BSA). Bars in the histograms represent mean values ± s.d., from technical replicates, with n = 3 independent dilutions of the cells.
